# The Manchester Royal Infirmary

**Published:** 1898-02-26

**Authors:** 


### The Institutional Workshop.

### THE MANCHESTER ROYAL INFIRMARY.

From the discussion in the City Council last week it seems very doubtful whether the people of Manchester will pay the price asked by the managers of the Infirmary for the ground by which it is surrounded. And, in truth, £286,000 is a large sum to pay for a certain amount of street widening, and we can hardly feel surprised that when the proposal of the committee came before the City Council it was referred back for reconsideration. To those outside the various interests involved it seems that the matter really ought to resolve itself into this, namely, whether to sell the whole site and build elsewhere, or to retain the whole site and build upon it the best hospital which its area will allow. The proposal which has just been considered would undoubtedly give the managers a very handsome sum for the erection of a new building elsewhere, but it would leave in their hands a plot in Piccadilly which would be wastefully large for a mere accident and out-patient hospital, while it would be too small for the erection of a new and complete hospital of the size required. A proposal which has now been made, however, for the Council to acquire the whole site for the erection of a free library and an art museum is another matter. This would leave the infirmary managers absolutely free, so that if they could obtain a suitable and central site they could erect a new infirmary all in one, or if thought better they could go further afield with their main building and put up an outpatient and emergency hospital in the best available site within the precincts. We are somewhat sceptical, however, about either of these projects coming to anything. It is all very well to theorise, but when it comes to the point of men responsible to their electors voting £286,000 for street widening or paying £550,000 for a mere site for a public building we doubt whether it will be done, and it seems extremely likely that the problem before the managers will come back to the old point, namely, how best to utilise the present site for hospital purposes. The Manchester people, however, must remember that they cannot both eat their cake and have it, and if they refuse to buy the land they must in all justice allow the managers to use it for infirmary purposes to the best advantage, in other words they must be prepared to see the site occupied by a very high building and one covering a good deal of what at present is open space. Much has been said of late about the bearing of modern antiseptic methods on hospital construction, and it has been more especially urged that their application has rendered the pavilion system of building unnecessary, and in fact obsolete. We cannot hold with these views, which seem to us to depend upon a misconception of the real drift of modern surgery. There is, however, something to be said in regard to the bearing of artificial ventilation upon the planning of a hospital, and especially upon the distance which need be interposed between the blocks or pavilions. Nevertheless, even with the most perfect mechanical ventilation, natural light is essential, and in a dark and smoky city the wings even of a block building cannot be crowded together. Probably the most perfect mechanically-ventilated hospital we have at the present time is the General Hospital at Birmingham, but, for all its mechanical ventilation, it is in every essential a pavilion hospital, and its wards are lighted on both sides.

Although it is true that mechanical ventilation may very much lessen the difficulties in the way of putting a large number of wards on a small space, especially by lessening the disadvantages commonly attaching to lofty buildings, it is by no means likely to justify the blocking of wards together in the manner to which the people of Manchester have been so long accustomed.

